# Walking is regulated by environmental temperature

**DOI:** 10.1038/s41598-021-91633-1

**Published:** 2021-06-09

**Authors:** Shuichi P. Obuchi, Hisashi Kawai, Juan C. Garbalosa, Kazumasa Nishida, Kenji Murakawa

**Affiliations:** 1 Tokyo Metropolitan Institute of Gerontology, 35-2 Sakae-cho, Itabashi-Ku, Tokyo, 173-0015 Japan; 2 Quinnipiac University, 275 Mount Carmel Avenue, Hamden, CT 06518 USA; 3 Taiyo Life Insurance Company, Tokyo, 2-7-1 Nihonbashi, Chuo-Ku, Tokyo, 103-6031 Japan

**Keywords:** Evolution, Physiology, Neurology

## Abstract

The mechanisms that regulate human walking are not fully understood, although there has been substantial research. In our study, we hypothesized that, although walking can be volitionally modified, it is also involuntary and controlled by evolutionary factors, such as the relationship between temperature and movement speed in poikilotherms. This study aimed to determine the effects of environmental temperature on speed, step length, and cadence during unrestrained walking over long periods. Customers of a private insurance company were asked to use a background smartphone GPS application that measured walking parameters. Participants were 1065 app users (298 men and 767 women) aged 14–86 years. Observed walking speed and cadence were higher in winter (average maximum temperature: 10.2 °C) than in summer (average maximum temperature: 29.8 °C) (p < 0.001). The walking parameters were closely related to environmental temperature, with cadence most strongly correlated with daily maximum temperature (r = − 0.812, p < 0.001) and indicating a curvilinear relationship. A decrease in environmental temperature was found to increase cadence when the temperature was below 30 °C. The findings suggest that walking may be regulated by environmental temperature and potentially by the autonomic nervous system’s response to environmental temperature.

## Introduction

Walking parameters, such as speed, step length, and cadence, are affected by various factors^1^. The most extensive research conducted on determinants of walking has been on the related energy costs^2,3^. Once a preferred walking speed that minimizes energy cost is selected, step length and cadence are automatically set as the optimal walk ratio^4,5^. Environmental factors have also been studied as the context for behavioural effects. For instance, walking speed is typically faster in urban areas than in rural areas^6,7^. Although they did not control for differences in demographics, Finnis and Walton^8^ found a logarithmic relationship between a city’s population size and walking speed. Environmental noise^9^, landscape^10^, and walking surface^11^ have also been shown to affect preferred walking speed.

Kimura et al.^12^ and Liang et al.^13^ suggested the effects of environmental temperatures on walking speed in humans. Kimura et al.^12^ noted that their participants exhibited a faster walking speed in winter than in summer, while Liang et al.^13^ reported a relationship between environmental temperature and walking speed during winter in locations with colder climates, and considered that low temperatures might be related to behaviour, in relation to wanting to reach their destination early. Levine and Norenzayn^14^ compared pedestrian walking in 31 countries and found that walking speed is faster in cities with cooler temperatures than in warmer cities. These results suggest that walking speed may be affected by environmental temperature; however, the conclusions of the previous studies were drawn using very limited research designs. For example, Kimura et al.’s study was not designed to investigate the relationship between walking and temperature, but only suggested the possibility that walking speeds may differ between winter and summer^12^. Liang et al. measured the cold season in one very low-temperature area (0 to – 30 °C) with unidentified participants, and found that people with slow walking speeds, such as older adults and children, selectively refrained from going out on cold days, resulting in an apparent increase in walking speed^13^. Levine and Norenzayn roughly examined the relationship between walking and some measurable environmental factors in 31 metropolitan areas, without any adjustment for covariates^14^. Thus, there is a lack of sufficient findings regarding whether walking is regulated by environmental temperature. Recent research on mice revealed that walking rhythms are regulated by midbrain activity, although walking parameters can be arbitrarily modified by high-level brain activity^15^. The effect of temperature on walking speed in such reports may be related to fundamental physiological controls, rather than behavioural effects.

In humans, the technical requirements of measurements make the effects of environmental temperature on walking parameters difficult to determine. In most studies, measurements of walking parameters have been conducted in controlled laboratory settings^16–19^. In this method, participants must visit the laboratory, and skilled technicians are required to take the measurements. Further, participants in laboratory settings may intentionally change their walking speed, and it is difficult to examine the effect of environmental temperature on walking parameters over longer periods of time. However, current technologies make it possible to investigate the effects of temperature on walking parameters. Specifically, GPS devices built into smartphones can reasonably measure walking speed, cadence, and step length in daily life over time^20,21^.

Changes in meteorological conditions can be easily observed in Japan, making it a suitable country for investigating the effects of meteorological changes on walking parameters. Japan is located at mid-latitudes with considerable altitude variations within the country. As a result, it has four distinct seasons and wide differences in temperature. For example, when we compare the temperature in Sapporo, the largest northern city, with that of Okinawa, the largest southern city, there is a 20 °C difference in winter and a 5 °C difference in summer in an average year. Additionally, in Sapporo, the difference in the average temperature between winter and summer is 27 °C, as compared to Okinawa where the difference is 12 °C^22^.

It has been reported that environmental temperature affects behaviour in poikilotherms; however, homeotherms, such as humans, may also have a similar primitive regulatory system^23^. We hypothesized that, although walking can be volitionally modified, there are involuntary controls that reflect human evolution, and walking is affected by environmental temperature. The purpose of this study was to determine the effects of environmental temperature on walking parameters during unrestrained walking measured over long periods of time. An understanding of the effects of environmental temperature on walking would be useful as part of the development and refinement of cardiovascular exercise programs that incorporate walking as a component.

## Results

**Figure 1:**
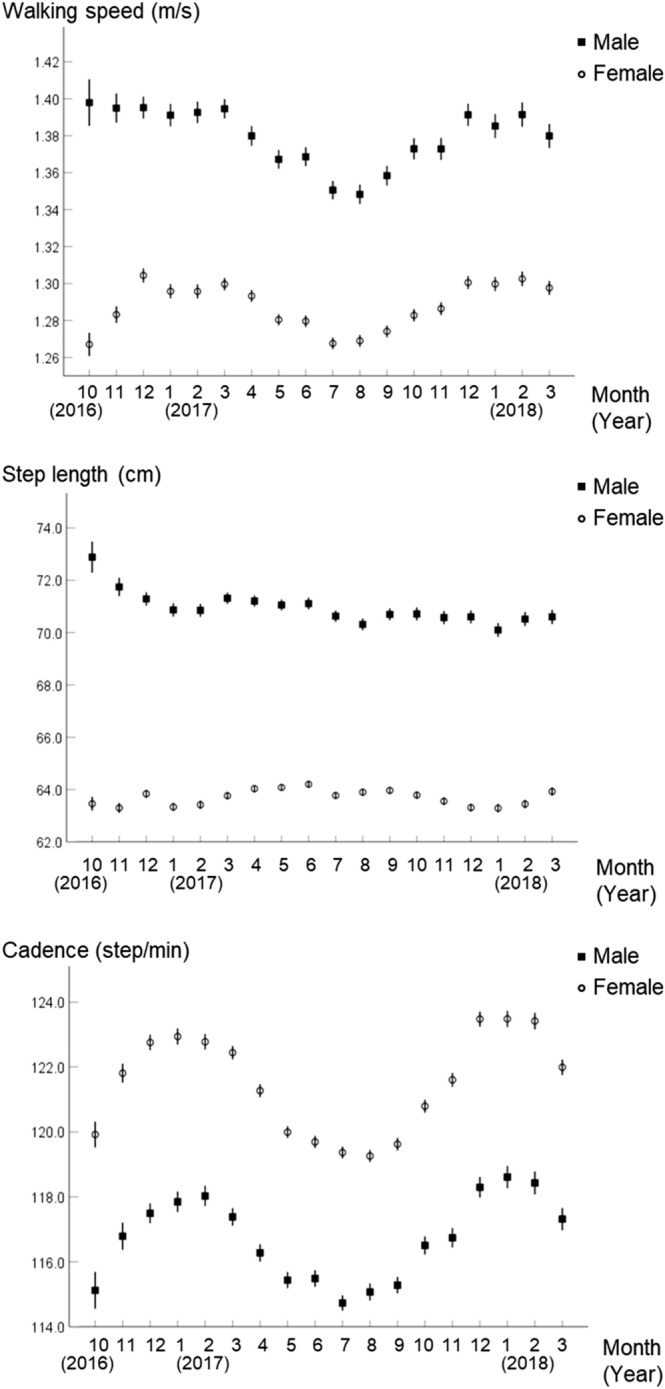
Seasonal changes in walking speed, step length, and cadence (error bar: 95% confidence interval, N = 1065).

The average (± 1 standard deviation) ages for men and women in our sample were 49 (± 14) and 52 (± 12) years, respectively. Changes in average walking speed, step length, and cadence for all the measured periods are shown in Fig. 1. Mean walking speed, step length, and cadence varied by month. Walking speed and cadence were significantly higher in winter and lower in summer, whereas seasonal changes in step length were small compared to those in walking speed and cadence. The effect sizes of walking speed, stride length, and cadence between summer and winter were 0.27, 0.00, and 0.48 for men and 0.33, − 0.09, and 0.58 for women, respectively (Table 1).

**Table 1:** Comparison of walking parameters between seasons.

	Spring				Summer				Autumn				Winter				Cohen’s d between summer and winter
	Mean	SD	Median	IQR	Mean	SD	Median	IQR	Mean	SD	Median	IQR	Mean	SD	Median	IQR	
**Male**																	
Walking speed (m/s)	1.37	0.11	1.37	1.290–1.45	1.35	0.11	1.35	1.27–1.42	1.36	0.11	1.37	1.28–1.43	1.38	0.11	1.39	1.31–1.46	0.27
Step length (cm)	70.6	5.1	70.4	67.3–73.9	70.4	5.0	70.6	67.2–73.6	70.4	5.1	70.5	67.1–74.2	70.3	5.1	70.5	66.8–74.1	0.00
Cadence (step/min)	116.5	6.3	115.8	112.1–120.3	114.9	6.0	114.7	110.7–118.4	115.7	6.1	115.3	111.5–119.3	118.0	6.6	117.7	113.4–122.0	0.48
**Female**																	
Walking speed (m/s)	1.28	0.09	1.28	1.23–1.34	1.26	0.09	1.26	1.20–1.31	1.27	0.09	1.27	1.21–1.33	1.29	0.09	1.29	1.23–1.35	0.33
Step length (cm)	63.7	4.1	63.6	61.1–66.5	63.6	4.2	63.5	61.1–66.3	63.6	4.2	63.6	60.9–66.5	63.3	4.2	63.1	60.6–66.2	− 0.09
Cadence (step/min)	121.0	6.6	121.1	116.6–124.8	118.9	6.2	119.0	114.7–122.7	120.3	6.6	120.3	116.2–124.3	122.8	7.0	122.8	118.1–127.1	0.58

*IQR* interquartile range, Linear mixed model and Bonferroni post-hoc test.

Male: Not significant: All seasons in step length, others: P < 0.05.

Female: Not significant: spring vs. summer, spring vs. autumn, summer vs. autumn in step length, others: P < 0.01.

Stratified analysis by age showed that the effect sizes of walking parameters between summer and winter tended to be similar to those found for the overall results; however, step length decreased in winter compared to summer in women aged 65 years and over (Supplementary Table 1, Supplementary Figs. 1–3).

The partial correlation coefficients of walking speed with all meteorological variables were statistically significant, and the correlation coefficient with maximum temperature was highest at − 0.518 (Table 2). For cadence, similar correlations were found between walking speed and meteorological variables, and the correlation coefficient with maximum temperature was − 0.812. The correlation coefficient for step length was slightly lower at 0.127. Stratified analysis by age group showed that the partial correlation coefficients of walking parameters with all meteorological variables tended to be similar to those of the overall results (Supplementary Tables 2–4).

**Table 2:** Walking parameters and meteorological variables correlation coefficients.

	Walking speed	Step length	Cadence
**Average temperature**			
Coefficient	− 0.505	0.131	− 0.798
Significance	< 0.001	< 0.001	< 0.001
df	1091	1091	1091
**Maximum temperature**			
Coefficient	− 0.518	0.127	− 0.812
Significance	< 0.001	< 0.001	< 0.001
df	1091	1091	1091
**Minimum temperature**			
Coefficient	− 0.482	0.132	− 0.768
Significance	< 0.001	< 0.001	< 0.001
df	1091	1091	1091
**Average humidity**			
Coefficient	− 0.238	0.048	− 0.361
Significance	< 0.001	0.111	< 0.001
df	1091	1091	1091
**Average air pressure**			
Coefficient	0.118	− 0.048	0.210
Significance	< 0.001	0.110	< 0.001
df	1091	1091	1091
**Average daylight hours**			
Coefficient	− 0.126	− 0.070	− 0.105
Significance	< 0.001	0.020	0.001
df	1091	1091	1091
**Average total solar radiation**			
Coefficient	− 0.306	0.030	− 0.441
Significance	< 0.001	0.329	< 0.001
df	1091	1091	1091

Control variable: gender.

**Figure 2:**
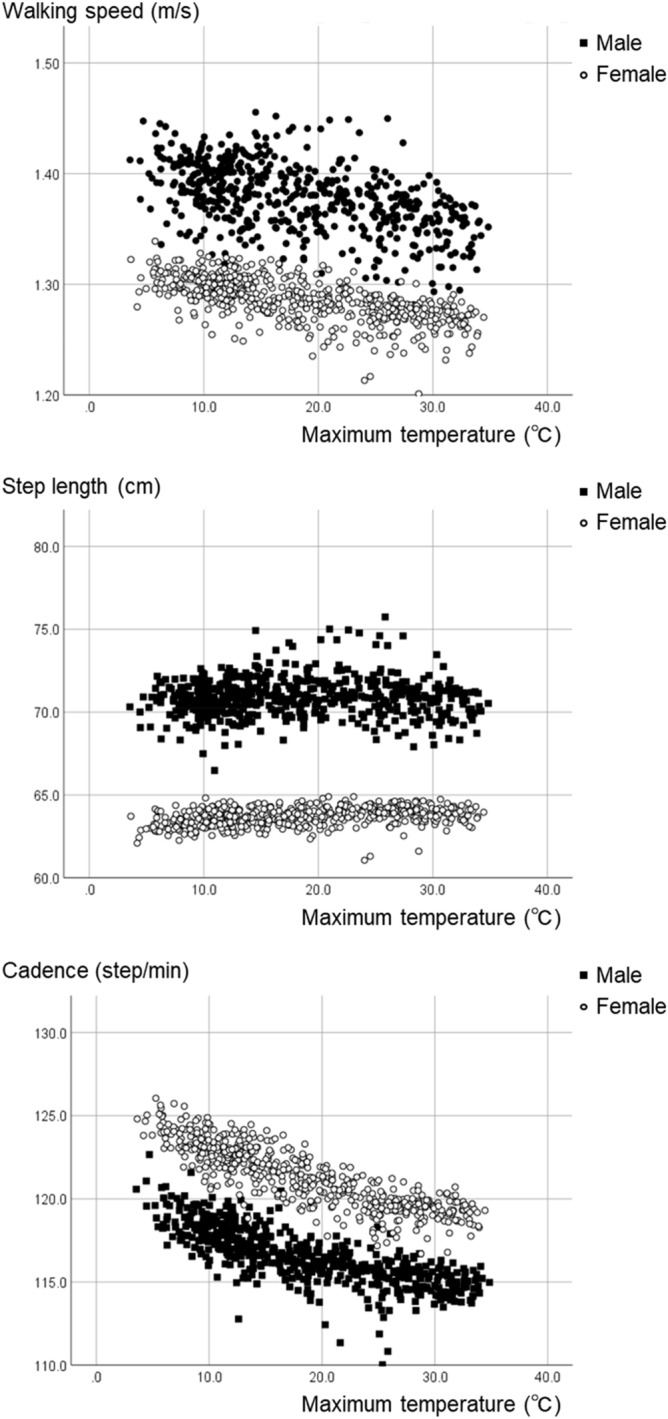
Scatter plots of the maximum temperatures and walking parameters.

Scatter plots of the maximum temperature and walking parameters are shown in Fig. 2. Maximum temperature showed an inverse correlation with walking speed and cadence, and the relationship was curvilinear. A decrease in environmental temperature increased cadence when the temperature was below 30 °C. Stratified analysis by age group showed that the scatter plots of the maximum temperature and walking parameters tended to be similar to those of the overall results (Supplementary Fig. 4).

## Discussion

Our research aimed to determine the effects of meteorological conditions on walking parameters in a mid-latitude country by observing unrestrained walking over one year. Although several studies have suggested that temperature decreases are related to faster walking speeds, no study to date has directly observed the relationship between seasonal climate variations and walking parameters.

We found that walking speed was faster in winter than in summer, with a difference of 0.03 m/s. This difference was smaller than the minimal detectable difference with a 95% confidence interval of 0.101 m/s reported in our previous study for change of daily life walking speed^20^. Thus, although the change may not be clinically significant, the effect sizes between summer and winter were approximately 0.3 and 0.5 for walking speed and cadence, respectively, which were regarded as a statistically medium to large change. Our results also suggested that this seasonal fluctuation in walking speed is mainly dependent on cadence, not step length, since the seasonal change of step length was smaller than that of cadence. Although walking parameters were significantly different between men and women and among age groups, the seasonal change was similar between genders and among age groups. In women aged 65 and older, the decrease in step length in winter compared to summer may have been caused by data in the winter of 2016, shortly after introducing and using the application.

To clarify which meteorological variable more greatly influenced walking, we investigated the relationships between temperature, humidity, barometric pressure, daylight hours, and cadence. We found that temperature was most closely related to cadence, although other variables were also significantly correlated. The scatter plots revealed inverse correlations between maximum temperatures, as recorded by the local weather station, and cadence, which was similar to curvilinear. When the temperature was below approximately 30 °C, cadence increased up to the lowest temperature (− 7.6 °C in the present data). This finding may suggest that walking could act as a heat production mechanism in humans under low or moderate temperature conditions. When the temperature becomes excessively high, the mechanism controlling cadence will cease to function.

An increase in cadence in winter will lead to an increase in the number of steps taken while walking. However, previous studies that examined seasonal changes in number of steps reported that steps decreased in winter compared to summer^24,25^. However, Yasunaga et al.^24^ reported that an amount of physical activity > 3 METs was conversely lower in summer than in winter, suggesting the possibility that exercise > 3 METs was reduced selectively to reduce body-heat production and maintain thermal equilibrium. The cadence response to environmental temperature may contribute slightly to maintaining decreased physical activity in winter due to taking fewer steps. In addition, the seasonal effects of temperature may impact not only cadence but may also have ramifications on the metabolic demands placed upon the body^26,27^. Given that walking is frequently included as a part of exercise regimens to help control A1C levels in patients with diabetes and for weight management in obese populations, health professionals may want to consider the impact of temperature on these metabolic demands.

For humans to maintain an appropriate body temperature, there are two mechanisms that directly affect skeletal muscles under cold temperature conditions to increase body temperature^28^. These heat-production mechanisms can be divided into non-shivering and shivering mechanisms. In particular, the shivering mechanism causes involuntary rhythmic contractions in skeletal muscles and is likely to share central pattern generators in the nervous system required for individuals to walk with a constant rhythm^29,30^. We assume that this autonomic reaction to cold temperatures can affect the seasonal fluctuation of cadence observed in the present study.

Sekiya et al.^4,5^ reported that when people increase walking speed voluntarily, although cadence and step length increase together, walk ratio (step length/cadence, m/steps/min) is kept at 0.006; walk ratio is controlled by the central nervous system to minimize the physiological cost of walking. The correlation analysis in the present study revealed that cadence and step length may be affected independently by temperature; thus, seasonal changes could alter the walk ratio. The walk ratio in this study increased slightly with maximum temperature and may be a result of changes in energy consumption caused by temperature variations. Our study results contradicted Sekiya et al.’s theory and revealed that, in winter, increases in walking speed are solely due to increases in cadence, suggesting variation in the walk ratio. Our results supported the idea that walking may be part of the autonomic system of thermoregulation.

GPS location estimation errors could be considered a possible factor related to seasonal fluctuations in walking parameters. The accuracy of GPS measurements is affected by tropospheric delays, higher-order effects of ionospheric delays, and ground deformations due to non-tidal ocean loading^31^. The Geospatial Information Authority of Japan confirms that there is a shift towards the west in winter, especially in northern Japan, due to tropospheric delays. However, out mean error was 2.51 mm, and the changes in walking speed we observed were larger than the GPS error. Additionally, the seasonal fluctuations in walking speed we observed were more dependent on changes in cadence, and thus not likely to be affected by GPS errors. Further, since the app used in this study identified walking from multiple positioning data, we believe that the effect of the GPS error was small. Our previous study reported no systematic errors in test–retest reliability at weekly intervals^20^; however, we are not confident that systematic errors will not be present over a long-term measurement period.

A limitation of our study is that GPS measurements did not provide precise walking conditions in each measurement; thus, we cannot neglect the effects of pedestrian conditions. Rainy or snowy pedestrian conditions may cause seasonal fluctuations in walking. Although we could not examine the effects of differences in walking conditions on walking speed, in our previous study^21^ we confirmed that walking speeds measured multiple times in daily life have a unimodal normal distribution, and that the average value is representative of typical daily walking speeds. We believe that this study assessed the participants’ representative walking speed, and that differences in walking conditions would have little impact on the study’s findings, since our results were based on long-term data collection and the reported average values were obtained using multiple collection periods. In addition, as we did not collect the measurement locations for privacy reasons, meteorological variables were based on the prefecture of residence for each participant. Hence, if participants walked in a location outside their residence, the meteorological variables and walking parameters would no longer match. However, according to data from the Statistics Bureau of the Ministry of Internal Affairs and Communications of Japan, the annual relocation rate between prefectures is 0.02%; thus, we believe that this issue would have almost no impact on the results of this study. Our data are also secondary use of the application’s primary uses; thus, we could not determine the effects of medical or other social conditions on seasonal fluctuations in walking.

Although it has been previously suggested that environmental temperature has an effect on walking, few studies have determined the direct effect of temperature on walking parameters. Our study observed this effect during unrestrained walking in various seasons with the help of a smartphone app. Our results indicated that walking is fundamentally regulated by environmental temperature through cadence control, although it is volitionally modified. The findings may suggest direct control of cadence by the autonomic nervous system.

## Methods

### Participants

At the time of first purchase or during a regular review of their health insurance policies, 166,579 subscribers to a private insurance service in Japan were asked to participate in the current study. All subscribers were asked to use a smartphone application that measured daily walking speed, step length, and step count. The software provided feedback to the customers in the form of bar graph displays that they could use to help them lead healthier lifestyles. The application also provided a ranking of users’ walking parameters.

Of the subscribers, 15,269 agreed to participate and provided informed consent. Informed consent was collected when the participants first used the smartphone application, and all participants provided their permission for walking parameter measurements to be collected for scientific research. If the application user was under 18 years of age, informed consent was obtained from their parent or guardian. Personal information and the exact GPS coordinates of their location were not recorded. If the participants required assistance downloading or using the application, insurance company employees helped them. Participants could also contact the insurance company by telephone if they had difficulties with the application during everyday use.

Walking parameters were measured from 1 October 2016 to 31 March 2018; 1065 (298 men and 767 women) participants, aged 14–86 years, who used the application for more than 12 consecutive months and could provide walking parameter measurements at least once a month were included in our study’s final analysis. All participants voluntarily provided their age, gender, and city of residence when they first used the application. We excluded data from individuals who did not provide this basic information from our final analysis. The final sample included participants in all prefectures in Japan except for Tottori Prefecture.

This study was carried out in accordance with the Declaration of Helsinki and approved by the institutional review board and ethics committee of the Tokyo Metropolitan Institute of Gerontology (Acceptance No. K128, 2018).

### Measurement of walking parameters

The application automatically measured walking speed, step length, cadence, and step count per day, in a manner that the participants did not notice. The walking start time was determined by the response of the pedometer application programming interface (API) in the smartphone operating system (OS) to reduce battery consumption relative to use of the accelerometer and geomagnetic sensors installed in the smartphone. The algorithms for determining walking initiation in each API are not published.

When a stable walking trajectory greater than or equal to 20 m was detected by the pedometer API and GPS, the walking speed was measured until it was interrupted. This application also measured participants’ walking cycle and step length using the pedometer API. Step length was derived by dividing the walking distance by the number of steps; it was not calculated from the measurement of each step, but was the average step length in the walking distance. The data for each continuous stretch of walking greater than or equal to 20 m were sent to the data server once a day, with the time for each measurement along with steps per day delivered from the pedometer API. For any incomplete 20 m stretch, the application did not store the walking parameters. The application works on both iOS and Android and is available for download from both the Apple Store and Google Play. The developer of the application stated that accuracy among smartphone models was confirmed with currently available smartphones (September 2016), but the data were not disclosed to the public.

Since the measurements of walking speed and step length were dependent on GPS, individuals who could not or did not walk outside for more than 20 m, that is, frail persons and house-ridden persons, were not included in our study. The accuracy of GPS walking measurement is reported elsewhere^32,33^. The validity and test–retest reliability of the application were reported in our previous study^20^.

### Meteorological variables

The average temperature, maximum and minimum temperatures, average humidity, average air pressure, average daylight hours, and average total solar radiation for each day according to the weather station nearest to the prefectural office were collected from the Japan Meteorological Agency^22^.

### Statistical analysis

All walking parameter measurements were aggregated by the participant’s identification number and measurement month, and changes in average walking speed, step length, and cadence for all the measured periods are shown in scatter plots. The measured periods were divided into four seasons: spring (March to May), summer (June to August), autumn (September to November), and winter (December to February). Linear mixed model and Bonferroni post-hoc test with walking speed, step length, and cadence as dependent variables and seasons as factors were conducted to determine seasonal effects on walking parameters. To assess effect sizes of the differences in each walking parameter between summer and winter, Cohen’s d between summer and winter was calculated.

For each day, the meteorological data for each user were averaged according to gender. This was combined with the walking parameter data aggregated by measurement day. Using these data, partial bivariate correlations were examined with gender as a control variable to assess the correlations between the walking parameters and meteorological variables. Scatter plots of the maximum temperature and walking parameters were depicted. The participants were divided into three age groups, under 40 years (83 men and 122 women), 40–64 years (170 men and 524 women), and 65 years and over (45 males and 121 females), and a stratified analysis by age group was also performed. The statistical significance level was set to 5%. All statistical analyses were conducted by SPSS ver. 24J. (IBM Japan, Ltd., Tokyo, Japan).

## Supplementary Information

Supplementary Figures.

Supplementary Tables.

## Data Availability

The datasets analysed in this study are not publicly available due to intellectual property rights, but are available upon reasonable request.
