# Supplementary material for: Walking is regulated by environmental temperature
Source: Sci Rep. 2021 Jun 9;11:12136. doi: 10.1038/s41598-021-91633-1 (PMC8190034; doi:10.1038/s41598-021-91633-1)
Supplement: Supplementary file 1 — Supplementary Figures. [file 41598_2021_91633_MOESM1_ESM.docx]

Q
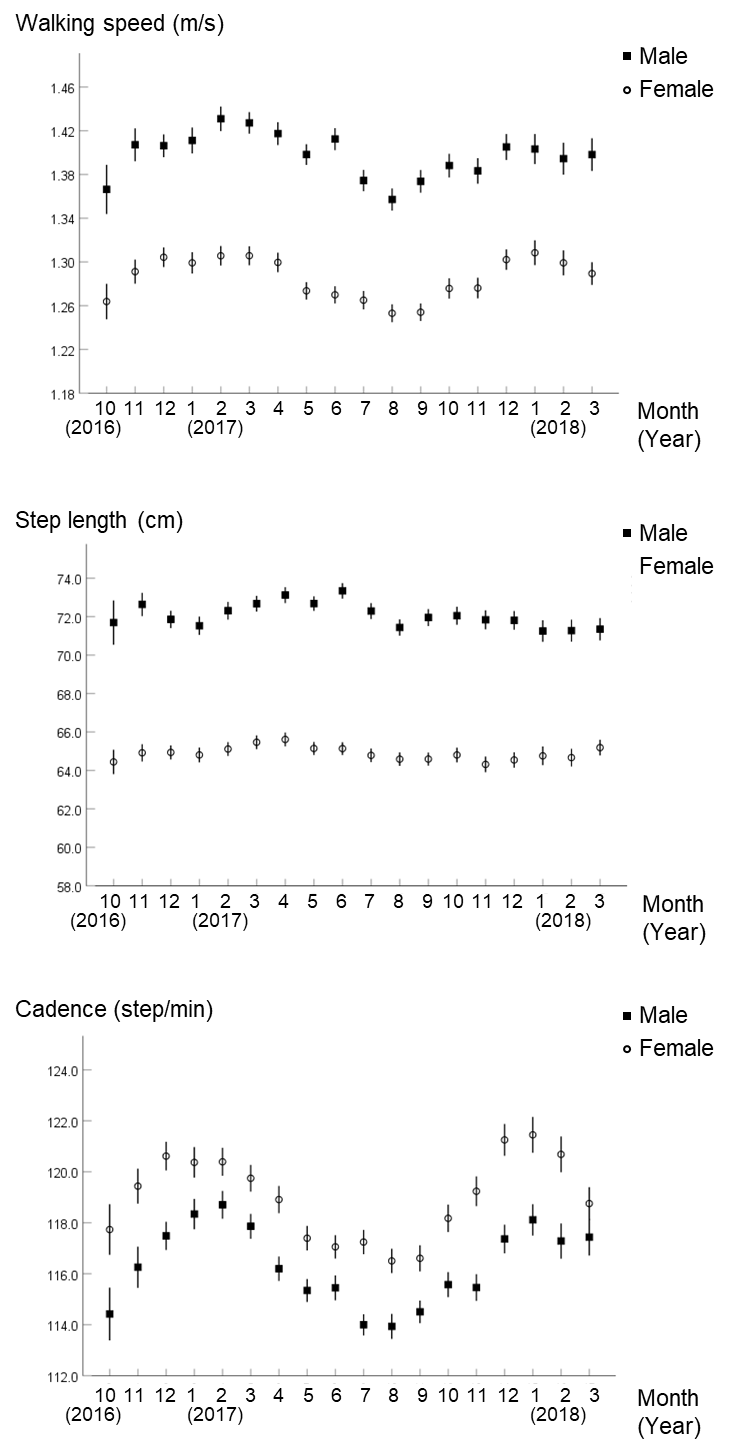


**Supplementary Figure 1.** Seasonal changes in walking speed, step length, and cadence in the under-40-years group (N = 205).


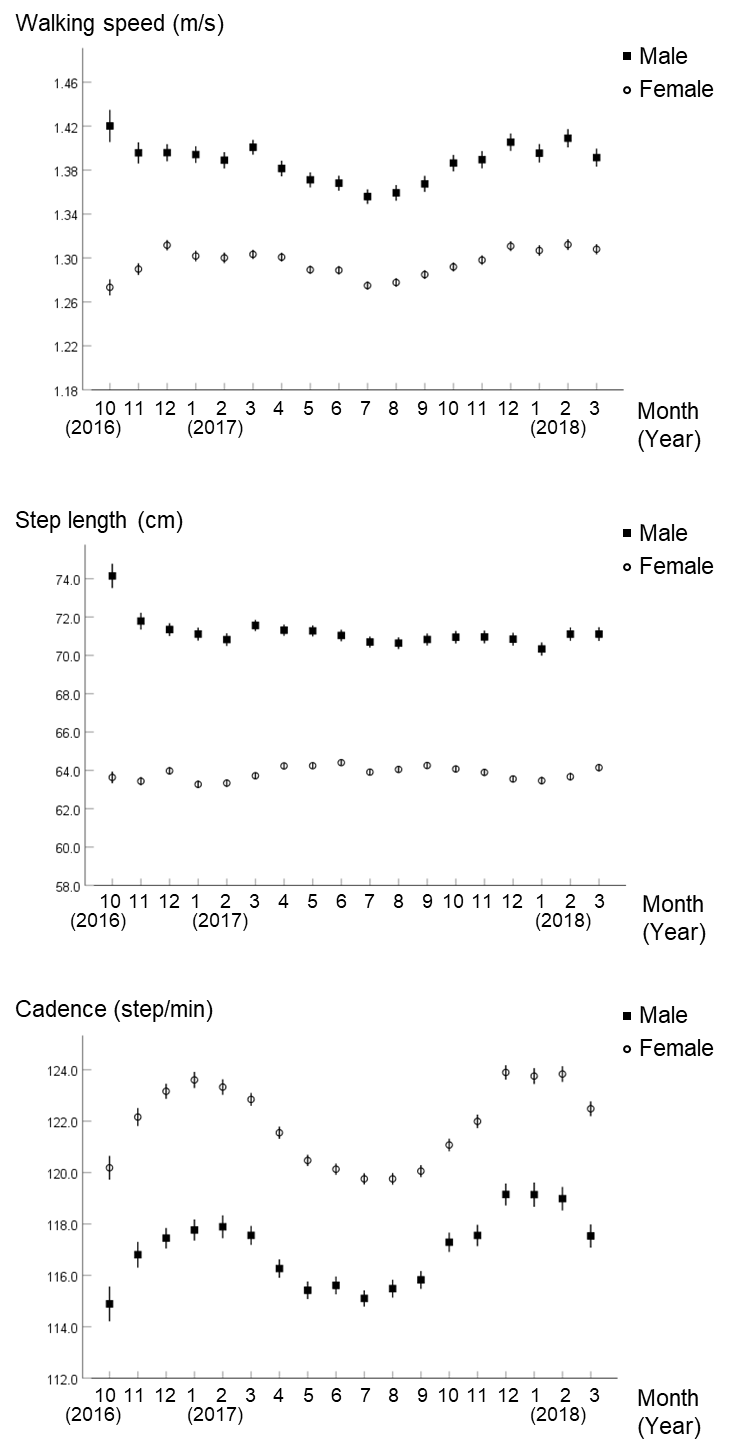


**Supplementary Figure 2.** Seasonal changes in walking speed, step length, and cadence in the 40–64-years group (N = 694).


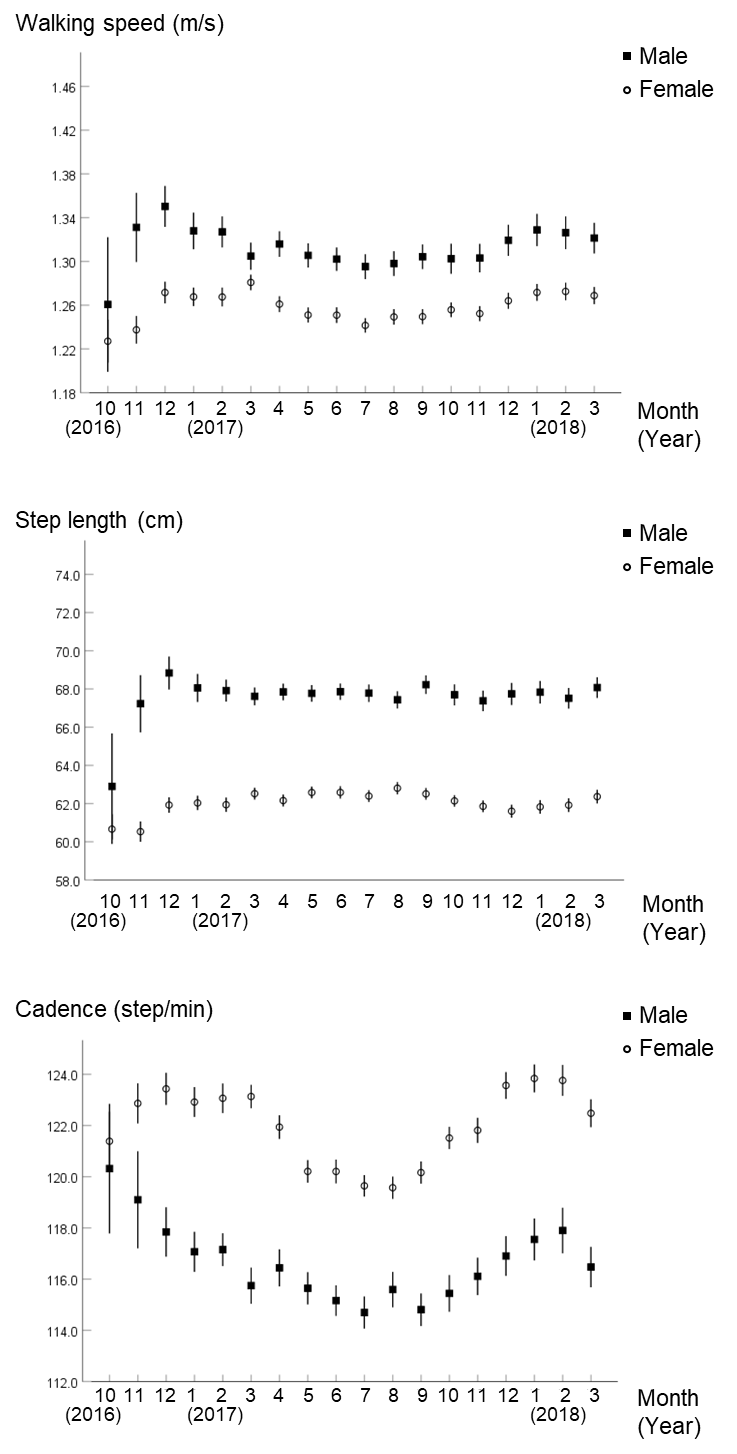


**Supplementary Figure 3.** Seasonal changes in walking speed, step length, and cadence in the 65-years-and-over group (N = 166).


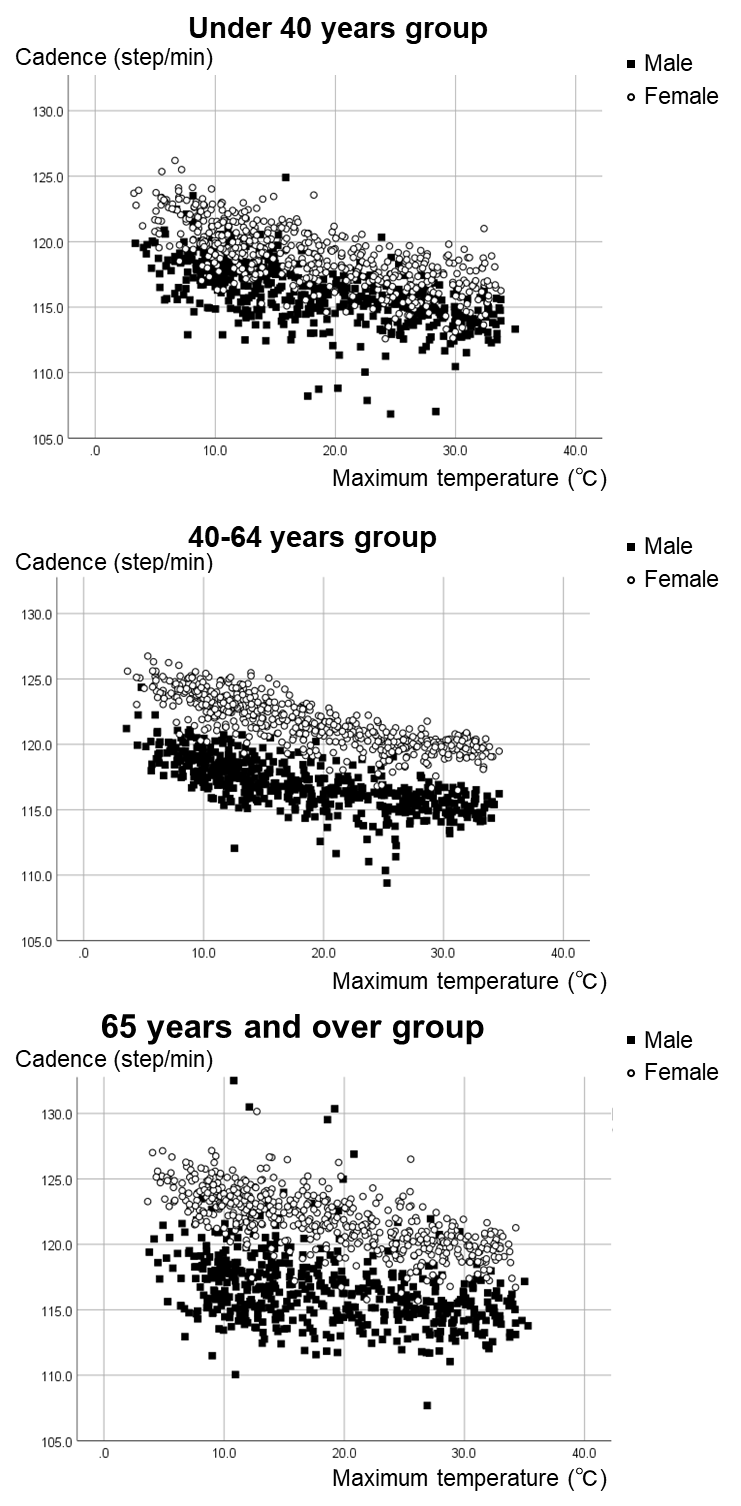


**Supplementary Figure 4.** Scatter plots of maximum temperature and cadence among the three age groups.
