# Supplementary material for: Walking is regulated by environmental temperature
Source: Sci Rep. 2021 Jun 9;11:12136. doi: 10.1038/s41598-021-91633-1 (PMC8190034; doi:10.1038/s41598-021-91633-1)
Supplement: Supplementary file 2 — Supplementary Tables. [file 41598_2021_91633_MOESM2_ESM.docx]

**Supplementary Table 1.** Comparison of walking parameters between seasons according to age group

| Male | |  |  |  |  |  |  |  |  |  |  |  |  |  |  |  |  |  |  |  |  |  |  |  |  |  |
| --- | --- | --- | --- | --- | --- | --- | --- | --- | --- | --- | --- | --- | --- | --- | --- | --- | --- | --- | --- | --- | --- | --- | --- | --- | --- | --- |
|  |  | Spring | | | | | | Summer | | | | | | Autumn | | | | | | Winter | | | | | | Cohen’s d between summer and winter |
|  |  | Mean | SD | Median | IQR | | | Mean | SD | Median | IQR | | | Mean | SD | Median | IQR | | | Mean | SD | Median | IQR | | |  |
| Under 40 years | |  |  |  |  |  |  |  |  |  |  |  |  |  |  |  |  |  |  |  |  |  |  |  |  |  |
|  | Walking speed (m/s) | 1.40 | 0.11 | 1.41 | 1.32 | - | 1.48 | 1.37 | 0.11 | 1.37 | 1.31 | - | 1.44 | 1.38 | 0.11 | 1.38 | 1.29 | - | 1.45 | 1.40 | 0.11 | 1.41 | 1.34 | - | 1.48 | 0.27 |
|  | Step length (cm) | 72.28 | 4.63 | 72.32 | 68.81 | - | 75.58 | 71.7 | 4.7 | 72.4 | 69.2 | - | 74.8 | 71.7 | 4.9 | 71.4 | 67.8 | - | 76.0 | 71.7 | 4.9 | 72.5 | 68.7 | - | 75.3 | -0.01 |
|  | Cadence (step/min) | 116.3 | 5.6 | 115.8 | 112.4 | - | 119.1 | 114.5 | 5.5 | 114.5 | 110.8 | - | 117.2 | 115.3 | 5.3 | 114.9 | 111.6 | - | 117.3 | 117.6 | 5.7 | 117.2 | 113.8 | - | 120.8 | 0.54 |
| 40−64 years | |  |  |  |  |  |  |  |  |  |  |  |  |  |  |  |  |  |  |  |  |  |  |  |  |  |
|  | Walking speed (m/s) | 1.37 | 0.11 | 1.37 | 1.29 | - | 1.45 | 1.35 | 0.10 | 1.35 | 1.27 | - | 1.42 | 1.36 | 0.10 | 1.37 | 1.28 | - | 1.43 | 1.39 | 0.10 | 1.39 | 1.32 | - | 1.46 | 0.40 |
|  | Step length (cm) | 70.5 | 5.1 | 70.2 | 67.4 | - | 73.7 | 70.5 | 5.1 | 70.9 | 67.3 | - | 73.6 | 70.5 | 5.1 | 70.7 | 67.6 | - | 74.3 | 70.5 | 5.0 | 70.7 | 67.7 | - | 73.9 | -0.01 |
|  | Cadence (step/min) | 116.8 | 6.5 | 116.4 | 112.3 | - | 120.8 | 115.1 | 6.1 | 115.0 | 110.8 | - | 118.7 | 116.0 | 6.3 | 115.6 | 111.8 | - | 120.0 | 118.4 | 6.9 | 118.3 | 113.6 | - | 122.5 | 0.50 |
| 65 years and over | |  |  |  |  |  |  |  |  |  |  |  |  |  |  |  |  |  |  |  |  |  |  |  |  |  |
|  | Walking speed (m/s) | 1.30 | 0.11 | 1.30 | 1.24 | - | 1.39 | 1.29 | 0.10 | 1.30 | 1.21 | - | 1.38 | 1.30 | 0.11 | 1.31 | 1.23 | - | 1.37 | 1.31 | 0.11 | 1.31 | 1.24 | - | 1.40 | 0.19 |
|  | Step length (cm) | 67.7 | 4.6 | 67.7 | 65.6 | - | 70.5 | 67.7 | 4.1 | 67.6 | 65.1 | - | 69.6 | 67.8 | 4.9 | 68.0 | 63.5 | - | 71.2 | 67.4 | 4.7 | 66.8 | 64.7 | - | 70.8 | -0.06 |
|  | Cadence (step/min) | 115.6 | 6.6 | 115.1 | 111.5 | - | 119.3 | 114.8 | 6.3 | 114.0 | 110.4 | - | 119.1 | 115.1 | 6.4 | 114.8 | 110.7 | - | 118.5 | 117.0 | 7.0 | 117.3 | 112.4 | - | 121.1 | 0.34 |
|  |  | | | | | | | | | | | | | | | | | | | | | | | | | |
| Female | |  |  |  |  |  |  |  |  |  |  |  |  |  |  |  |  |  |  |  |  |  |  |  |  |  |
|  |  | Spring | | | | | | Summer | | | | | | Autumn | | | | | | Winter | | | | | | Cohen’s d between summer and winter |
|  |  | Mean | SD | Median | IQR | | | Mean | SD | Median | IQR | | | Mean | SD | Median | IQR | | | Mean | SD | Median | IQR | | |  |
| Under 40 years | |  |  |  |  |  |  |  |  |  |  |  |  |  |  |  |  |  |  |  |  |  |  |  |  |  |
|  | Walking speed (m/s) | 1.28 | 0.08 | 1.28 | 1.23 | - | 1.34 | 1.25 | 0.08 | 1.24 | 1.20 | - | 1.29 | 1.26 | 0.09 | 1.25 | 1.21 | - | 1.32 | 1.29 | 0.08 | 1.28 | 1.24 | - | 1.34 | 0.50 |
|  | Step length (cm) | 64.9 | 3.7 | 64.8 | 62.1 | - | 67.3 | 64.4 | 3.9 | 64.0 | 61.8 | - | 67.2 | 64.4 | 3.9 | 64.7 | 61.5 | - | 66.7 | 64.4 | 3.6 | 64.4 | 62.1 | - | 66.7 | 0.01 |
|  | Cadence (step/min) | 118.7 | 5.9 | 118.4 | 114.6 | - | 122.7 | 116.6 | 5.8 | 116.9 | 111.9 | - | 120.4 | 117.8 | 6.2 | 117.9 | 113.6 | - | 121.9 | 120.3 | 6.6 | 119.5 | 115.6 | - | 125.4 | 0.60 |
| 40−64 years | |  |  |  |  |  |  |  |  |  |  |  |  |  |  |  |  |  |  |  |  |  |  |  |  |  |
|  | Walking speed (m/s) | 1.29 | 0.09 | 1.29 | 1.23 | - | 1.35 | 1.27 | 0.09 | 1.27 | 1.21 | - | 1.32 | 1.28 | 0.09 | 1.28 | 1.22 | - | 1.34 | 1.30 | 0.09 | 1.30 | 1.24 | - | 1.36 | 0.33 |
|  | Step length (cm) | 63.8 | 4.1 | 63.5 | 61.4 | - | 66.5 | 63.8 | 4.1 | 63.7 | 61.4 | - | 66.3 | 63.8 | 4.1 | 63.6 | 61.3 | - | 66.7 | 63.4 | 4.2 | 63.2 | 60.8 | - | 66.4 | -0.09 |
|  | Cadence (step/min) | 121.4 | 6.6 | 121.4 | 117.1 | - | 125.3 | 119.3 | 6.2 | 119.3 | 115.3 | - | 123.0 | 120.7 | 6.5 | 120.5 | 116.7 | - | 124.5 | 123.2 | 7.0 | 123.5 | 118.5 | - | 127.4 | 0.59 |
| 65 years and over | |  |  |  |  |  |  |  |  |  |  |  |  |  |  |  |  |  |  |  |  |  |  |  |  |  |
|  | Walking speed (m/s) | 1.26 | 0.09 | 1.25 | 1.19 | - | 1.31 | 1.24 | 0.09 | 1.24 | 1.18 | - | 1.29 | 1.25 | 0.09 | 1.24 | 1.19 | - | 1.31 | 1.26 | 0.08 | 1.25 | 1.20 | - | 1.31 | 0.23 |
|  | Step length (cm) | 62.2 | 4.1 | 62.0 | 59.5 | - | 64.9 | 62.3 | 4.5 | 62.3 | 59.3 | - | 65.2 | 61.8 | 4.4 | 61.4 | 59.0 | - | 64.8 | 61.3 | 4.1 | 60.9 | 58.8 | - | 63.9 | -0.22 |
|  | Cadence (step/min) | 121.5 | 6.6 | 121.4 | 117.5 | - | 125.5 | 119.5 | 6.4 | 119.6 | 114.9 | - | 123.3 | 121.1 | 6.5 | 121.0 | 116.4 | - | 124.8 | 123.2 | 6.9 | 122.4 | 118.9 | - | 127.2 | 0.56 |

IQR: interquartile range

**Supplementary Table 2.** Walking parameters and meteorological variables correlation coefficients in the under-40-years group

|  | | Walking Speed | Step length | Cadence |
| --- | --- | --- | --- | --- |
| Average Temperature | Coefficient | -0.408 | 0.040 | -0.670 |
|  | Significance | 0.000 | 0.185 | 0.000 |
|  | df | 1078 | 1078 | 1078 |
| Maximum Temperature | Coefficient | -0.415 | 0.038 | -0.679 |
|  | Significance | 0.000 | 0.214 | 0.000 |
|  | df | 1078 | 1078 | 1078 |
| Minimum Temperature | Coefficient | -0.392 | 0.042 | -0.646 |
|  | Significance | 0.000 | 0.165 | 0.000 |
|  | df | 1078 | 1078 | 1078 |
| Average Humidity | Coefficient | -0.221 | -0.003 | -0.331 |
|  | Significance | 0.000 | 0.917 | 0.000 |
|  | df | 1078 | 1078 | 1078 |
| Average Air Pressure | Coefficient | 0.044 | -0.101 | 0.175 |
|  | Significance | 0.148 | 0.001 | 0.000 |
|  | df | 1078 | 1078 | 1078 |
| Average Daylight Hours | Coefficient | -0.056 | -0.019 | -0.071 |
|  | Significance | 0.064 | 0.531 | 0.020 |
|  | df | 1078 | 1078 | 1078 |
| Average Total Solar Radiation | Coefficient | -0.174 | 0.074 | -0.354 |
|  | Significance | 0.000 | 0.015 | 0.000 |
|  | df | 1078 | 1078 | 1078 |
| Control variable: gender |  |  |  |  |

**Supplementary Table 3.** Walking parameters and meteorological variables correlation coefficients in the 40−64-years group

|  | | Walking Speed | Step length | Cadence |
| --- | --- | --- | --- | --- |
| Average Temperature | Coefficient | -0.430 | 0.131 | -0.751 |
|  | Significance | 0.000 | 0.000 | 0.000 |
|  | df | 1091 | 1091 | 1091 |
| Maximum Temperature | Coefficient | -0.445 | 0.124 | -0.765 |
|  | Significance | 0.000 | 0.000 | 0.000 |
|  | df | 1091 | 1091 | 1091 |
| Minimum Temperature | Coefficient | -0.407 | 0.134 | -0.722 |
|  | Significance | 0.000 | 0.000 | 0.000 |
|  | df | 1091 | 1091 | 1091 |
| Average Humidity | Coefficient | -0.184 | 0.068 | -0.334 |
|  | Significance | 0.000 | 0.024 | 0.000 |
|  | df | 1091 | 1091 | 1091 |
| Average Air Pressure | Coefficient | 0.122 | -0.031 | 0.209 |
|  | Significance | 0.000 | 0.305 | 0.000 |
|  | df | 1091 | 1091 | 1091 |
| Average Daylight Hours | Coefficient | -0.142 | -0.105 | -0.092 |
|  | Significance | 0.000 | 0.000 | 0.002 |
|  | df | 1091 | 1091 | 1091 |
| Average Total Solar Radiation | Coefficient | -0.307 | -0.023 | -0.414 |
|  | Significance | 0.000 | 0.454 | 0.000 |
|  | df | 1091 | 1091 | 1091 |
| Control variable: gender |  |  |  |  |

**Supplementary Table 4.** Walking parameters and meteorological variables correlation coefficients in the 65-years-and-over group

|  | | Walking Speed | Step length | Cadence |
| --- | --- | --- | --- | --- |
| Average Temperature | Coefficient | -0.346 | -0.003 | -0.531 |
|  | Significance | 0.000 | 0.910 | 0.000 |
|  | df | 1062 | 1062 | 1062 |
| Maximum Temperature | Coefficient | -0.344 | 0.009 | -0.545 |
|  | Significance | 0.000 | 0.779 | 0.000 |
|  | df | 1062 | 1062 | 1062 |
| Minimum Temperature | Coefficient | -0.341 | -0.016 | -0.508 |
|  | Significance | 0.000 | 0.607 | 0.000 |
|  | df | 1062 | 1062 | 1062 |
| Average Humidity | Coefficient | -0.167 | -0.066 | -0.173 |
|  | Significance | 0.000 | 0.033 | 0.000 |
|  | df | 1062 | 1062 | 1062 |
| Average Air Pressure | Coefficient | 0.013 | -0.087 | 0.132 |
|  | Significance | 0.671 | 0.005 | 0.000 |
|  | df | 1062 | 1062 | 1062 |
| Average Daylight Hours | Coefficient | -0.017 | 0.090 | -0.139 |
|  | Significance | 0.586 | 0.003 | 0.000 |
|  | df | 1062 | 1062 | 1062 |
| Average Total Solar Radiation | Coefficient | -0.144 | 0.105 | -0.354 |
|  | Significance | 0.000 | 0.001 | 0.000 |
|  | df | 1062 | 1062 | 1062 |
| Control variable: gender |  |  |  |  |
